# Structural Analysis of the Synthetic Duffy Binding Protein (DBP) Antigen DEKnull Relevant for *Plasmodium vivax* Malaria Vaccine Design

**DOI:** 10.1371/journal.pntd.0003644

**Published:** 2015-03-20

**Authors:** Edwin Chen, Nichole D. Salinas, Francis B. Ntumngia, John H. Adams, Niraj H. Tolia

**Affiliations:** 1 Department of Molecular Microbiology and Microbial Pathogenesis, Washington University School of Medicine, Saint Louis, Missouri, United States of America; 2 Global Health Infectious Disease Research, Department of Global Health, College of Public Health, University of South Florida, Tampa, Florida, United States of America; 3 Department of Biochemistry and Molecular Biophysics, Washington University School of Medicine, Saint Louis, Missouri, United States of America; Federal University of São Paulo, BRAZIL

## Abstract

The *Plasmodium vivax* vaccine candidate Duffy Binding Protein (DBP) is a protein necessary for *P*. *vivax* invasion of reticulocytes. The polymorphic nature of DBP induces strain-specific immune responses that pose unique challenges for vaccine development. DEKnull is a synthetic DBP based antigen that has been engineered through mutation to enhance induction of blocking inhibitory antibodies. We determined the x-ray crystal structure of DEKnull to identify if any conformational changes had occurred upon mutation. Computational and experimental analyses assessed immunogenicity differences between DBP and DEKnull epitopes. Functional binding assays with monoclonal antibodies were used to interrogate the available epitopes in DEKnull. We demonstrate that DEKnull is structurally similar to the parental Sal1 DBP. The DEKnull mutations do not cause peptide backbone shifts within the polymorphic loop, or at either the DBP dimerization interface or DARC receptor binding pockets, two important structurally conserved protective epitope motifs. All B-cell epitopes, except for the mutated DEK motif, are conserved between DEKnull and DBP. The DEKnull protein retains binding to conformationally dependent inhibitory antibodies. DEKnull is an iterative improvement of DBP as a vaccine candidate. DEKnull has reduced immunogenicity to polymorphic regions responsible for strain-specific immunity while retaining conserved protein folds necessary for induction of strain-transcending blocking inhibitory antibodies.

## Author Summary

*Plasmodium vivax* is an oft neglected causative agent of human malaria. It inflicts tremendous burdens on public health infrastructures and causes significant detrimental effects on socio-economic growth throughout the world. *P*. *vivax* Duffy Binding Protein (DBP) is a surface protein that the parasite uses to invade host red blood cells and is a leading vaccine candidate. The variable nature of DBP poses unique challenges in creating an all-encompassing generalized vaccine. One method to circumvent this problem is to synthetically engineer a single artificial protein antigen that has reduced variability while maintaining conserved protective motifs to elicit strain-transcending protection. This synthetic antigen is termed DEKnull. Here, we provide structural and biochemical evidence that DEKnull was successfully engineered to eliminate polymorphic epitopes while retaining the overall fold of the protein, including conserved conformational protective epitopes. Our work presents validation for an improved iteration of the DBP *P*. *vivax* vaccine candidate, and provides evidence that protein engineering is successful in countering DBP polymorphisms. In doing so, we also lay down the foundation that engineering synthetic antigens is a viable approach and should be considered in future vaccine designs for pathogens.

## Introduction

*Plasmodium vivax* is a causative agent of malaria, inflicting significant morbidity and impeding economic growth in highly endemic areas [1,2]. Increasing evidence indicates the severity of disease, economic impact, and burden of *P*. *vivax* has been severely underestimated [1,2]. Among the proposed methods for disease control, vaccines are appealing for a multitude of reasons. Vaccines are cost-effective, efficient, and have been historically successful in combating infectious diseases especially in resource poor environments [3]. Individuals living in regions with *P*. *vivax* develop naturally acquired protective immunity and antibodies isolated from those naturally immune have anti-DBP inhibitory effects that correlate with results from *in vitro* functional assays [4–6].

Establishment of a successful host infection necessitates specific receptor-ligand interactions between host red blood cells and *Plasmodium* parasites [7]. For *P*. *vivax*, the critical interaction is that between the merozoite Duffy binding protein (DBP) and the Duffy antigen receptor for chemokines (DARC) on reticulocytes. DARC-negative individuals are resistant to clinical *P*. *vivax* infection, and naturally immune individuals can possess anti-DBP antibodies that inhibit the DBP-DARC interaction and prevent parasite growth [6,8–12]. Additionally, polyclonal antibodies elicited by recombinant DBP exhibit similar protective and inhibitive effects to naturally acquired antibodies [6,11,13,14]. Certain isolates of *P*. *vivax* have been reported to invade Duffy-negative cells [15]. However, sequencing of these isolates identified a gene encoding a DBP paralog suggesting the increased copy number and/or expression of DBP may enable invasion into Duffy-negative cells [16]. Together, this highlights the central importance of the DBP-DARC interaction in *P*. *vivax* infection and presents DBP as a crucial parasite protein that can be developed as a vaccine target.

DBP is a member of the Duffy binding-like erythrocyte binding protein (DBL-EBP) family, and binds DARC through a conserved cysteine-rich DBL domain known as region II (DBP-II) [17–22]. DBP-II engages DARC through a multimeric assembly mechanism where two DBP-II domains initially bind one DARC to form a heterotrimer that rapidly recruits a second DARC to form a heterotetramer [23–26]. DBP-II amino acids F261-T266, L270-K289, and Q356-K367 form critical contacts with the DARC ectodomain during this process [23]. This receptor-induced ligand-dimerization model is conserved amongst other members of the DBL-EBP family and provides spatial orientation for DBL domains at the parasite-RBC membrane interface [24–30]. Residues that mediate multimeric assembly are important targets of protective immunity as the epitopes of naturally acquired anti-DBP-II antibodies that disrupt the DBP-DARC interaction localize to residues at the dimerization interface, DARC binding pockets, and the RBC proximal face of DBP-II [10]. However, clusters of highly polymorphic residues flank these protective epitopes, which is a pattern seen in pathogens undergoing selective pressure that results in an immune evasion where allelic variants can escape immunity elicited by a previous infection [10,21,26,31–37]. Therefore, polymorphic residues of DBP appear to have a high potential to be the basis of strain specific immune responses that misdirects immune responses away from conserved targets of broadly neutralizing protection. Although strain specific immunity can be protective these seemingly more immunogenic epitopes offer limited value because of the strain-limited nature of the immunity. Genetic analysis of DBP-II alleles reveal a high *d*_N_/*d*_S_ ratio often seen when selection pressure drives allelic diversity as a mechanism for immune evasion [38–42]. In order to proceed with DBP as a *P*. *vivax* vaccine target, it is therefore critical to address the challenges presented by polymorphism and immune misdirection inherent in this allelic diversity.

**Fig 1:**
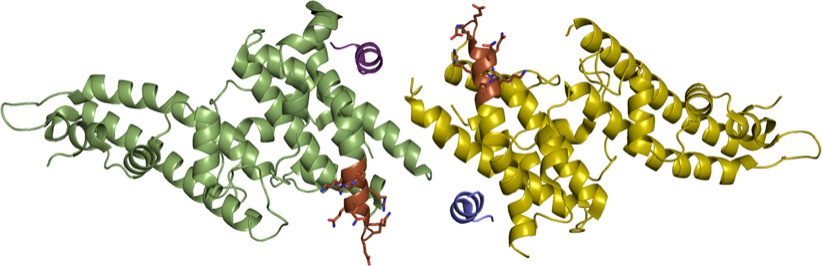
Location of DEK polymorphisms on Sal1 DBP-II. DEK polymorphisms (DEKAQQRRKQ) mapped onto the Sal1 DBP-II and DARC heterotetramer. DEK residues are shown in brown and side chains are displayed as sticks. The two DBP-II molecules are in green and yellow. The two DARC peptides are in purple and blue.

Immunization with DBP-II elicits weakly reactive and allele specific immune responses, a far cry from the end objective of inducing strain-transcending protection [38]. The poor protectivity appears to be due in part to polymorphic non-functional residues diverting the immune response away from the more conserved, less immunogenic, critical receptor binding residues [10,38,43–45]. Consistent with this view, the most polymorphic region, identified as the DEK epitope, is positioned immediately adjacent to the conserved DARC-binding groove (Fig. 1) [10,23]. Antibodies to the DEK epitope can disrupt DBP function, but inhibition is strain limited. Therefore, we refer to DEK as a decoy epitope that distracts the immune response away for more conserved functional epitopes that could serve as basis of a broadly neutralizing protective immunity. To overcome this inherent deficiency of DBP as an immunogen, a novel synthetic DBP-II antigen termed DEKnull was engineered where the polymorphic residues that comprise the DEK epitope were mutated to amino acids not usually present (Fig. 1, S1 Fig) [38]. These proof of principle studies demonstrated the feasibility of redirecting the immune response to conserved, critical residues by eliminating polymorphic epitopes with the goal to create a vaccine that induces a greater percentage of protective antibodies to more conserved, less immunogenic epitopes. Indeed, anti-DEKnull sera lost reactivity towards the polymorphic patch as predicted, but still retained the ability to generate inhibitory antibodies, including epitopes reactive to naturally-occurring immune antibodies of persons infected with *P*. *vivax* [38,46]. DEKnull also induced strong anamnestic responses that were protective and cross-reactive against a panel of different DBP-II alleles [5]. Furthermore, DEKnull produced a more consistent inhibitory profile across variants [46].

However, mutation can alter the three-dimensional structure of a protein that in turn would alter the available epitopes presented in a synthetic antigen. This study presents the structure of a synthetic *Plasmodium* antigen and its implications for the future of vaccine design in targeting malaria. We determined the structure of DEKnull to identify if any shifts in fold and secondary structure or sub-domain rearrangements had occurred, and whether these changes affect DEKnull’s potential as a vaccine surrogate for native alleles [26]. The effects of mutating the DEK polymorphic patch on conserved protective epitopes was identified by comparison with the pre-existing Sal1 structure [26]. We examined and compared the epitope profile of DEKnull to DBP-II using computational approaches as well as through interrogation with a panel of DBP monoclonal antibodies [47]. Together these studies inform future efforts to guide the rational design of the next iteration of a synthetic DBP-II antigen to improve its immunogenicity and ability to mount a thoroughly protective response.

## Materials and Methods

### Protein expression and purification

DEKnull was obtained by oxidative refolding. Inclusion bodies expressed in *E*. *coli* were solublized in 6 M guanidinium hydrochloride and refolded via rapid dilution in 400 mM L-arginine, 50 mM Tris pH 8.0, 10 mM EDTA, 0.1 mM PMSF, 2 mM reduced glutathione, and 0.2 mM oxidized glutathione. Refolded protein was captured on SP Sepharose Fast Flow resin (GE Healthcare), eluted with 50 mM MES pH 6.0, 700 mM NaCl, and dialyzed overnight in 50 mM MES pH 6.0, 100 mM NaCl. The protein was subsequently purified by sequential size exclusion chromatography (GF200) and ion exchange chromatography (HiTrapS). Protein was finally buffer exchanged into 10 mM HEPES pH 7.4, 100 mM NaCl with size exclusion chromatography. Sal1 DBP-II was purified similarly as DEKnull, but without overnight dialysis.

### Protein crystallization and data collection

DEKnull crystals were grown by hanging-drop vapor diffusion. First, 1 μL of protein solution at 3–9 mg/mL was mixed with 1 μL of reservoir containing 0.2 M di-sodium tartrate, 20% PEG 3350 to create needle clusters. Crystals were shattered and microseeded into a mix of 1 μL of protein solution at 4 mg/mL and 1 μL of reservoir containing 0.2 M lithium chloride, 20% PEG 3350. Large needle rods of DEKnull grew within a week and were flash frozen in liquid nitrogen. Data was collected to a resolution of 2.1 Å at beamline 4.2.2 of the Advanced light Source, Lawrence Berkeley National Laboratory and processed with XDS [48].

### Structure solution and analysis

The DEKnull structure was solved by molecular replacement in PHASER [49] using a single Sal1 DBP-II domain from 3RRC as a starting model. Manual rebuilding in COOT [50] and refinement in PHENIX led to a final refined model with final R-factor/R-free of 21.77%/25.88% with good geometry as reported by MOLPROBITY [50–52]. The MOLPROBITY score of 0.81 places this structure in the top 100^th^ percentile of structures 1.85–2.35 Å. 98.22% of residues lie in favored, 1.78% of residues lie in additionally allowed, and 0% lie in disallowed regions of the Ramachandran plot. Atomic coordinates and structure factors have been deposited into the Protein Data Bank with accession code 4YFS.

### ELISA assays with anti-DBP antibodies

The ELISAs were performed as previously described [28]. Briefly, BSA, Sal1 DBP-II, and DEKnull were coated on the plate overnight at 4°. The plates were washed with PBS/Tween-20 and then blocked with 2% BSA in PBS/Tween-20 for one hour at room temperature. The plates were washed with PBS/Tween-20 and then incubated with anti-DBP antibodies (2C6, 2D10, 2H2, 3C9, 2F12, 3D10) individually for one hour at room temperature. The plates were again washed with PBS/Tween-20 and then incubated with an anti-mouse secondary antibody conjugated to Alexafluro-488 for 30 minutes at room temperature. After a final wash step, the fluorescence was measured using a POLARstar Omega (BMG Labtech) plate reader.

## Results

### Structure of the synthetic DEKnull antigen

**Fig 2:**
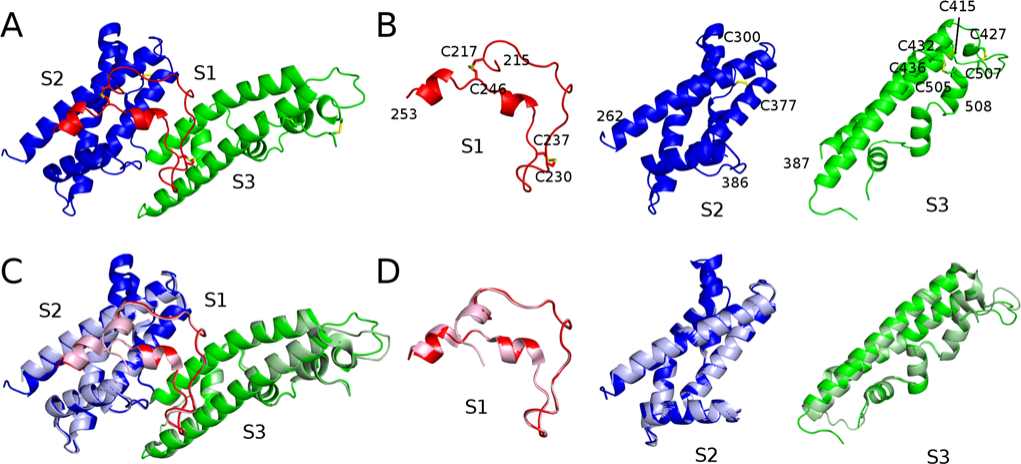
DEKnull is structurally similar to Sal1 DBP-II. (A) DEKnull separated into three sub-domains, sub-domain 1 (S1—red), sub-domain 2 (S2—blue), and sub-domain 3 (S3—green). (B) S1 (red) contains a β-hairpin, S2 (blue) is a helix bundle, and S3 (green) is a helix bundle. Domain boundaries and disulfide bonding cysteines are labeled. (C) Structural alignment of DEKnull (solid colors) with Sal1 DBP-II (light tinted colors) with r.m.s. deviation of 0.435Å. (D) Structural alignment of individual DEKnull sub-domains (solid colors) with Sal1 DBP-II sub-domains (light tinted colors). S1 alignment has a r.m.s. deviation of 0.308 Å. S2 alignment has a r.m.s. deviation of 0.288 Å. S3 alignment has a r.m.s. deviation of 0.310 Å.

We obtained the crystal structure of the DEKnull antigen to a resolution of 2.1 Å (Table 1). DEKnull maintains the overall fold and conserved disulfide bonding patterns of a DBL domain similar to that found in *P*. *vivax* DBP Sal1, from which DEKnull is derived [23,26]. The DBL fold is a conserved structural feature in other important *Plasmodium* adhesion proteins, including the *P*. *falciparum* EBA-175 and EBA-140, *P*. *knowlesi* α-DBP protein, and the NTS-DBL1α_1_, DBL6ε, and DBL3x domains of PfEMP-1 (Fig. 2A) [26,27,53–58]. DEKnull also retains the characteristic three sub-domain architecture of DBL domains with critical intra-domain disulfide bonding patterns (Fig. 2B). Sub-domain 1 (S1) contains residues K215 to L253 with two disulfide bonds, C217-C246 and C230-C237. Sub-domain 2 (S2) contains residues H262 to E386 and has a single disulfide bond C300-C377. Sub-domain 3 (S3) contains residues P387 to S508 and has three disulfide bonds: C415-C432, C427-C507, and C436-C505. All cysteines in DEKnull are involved in disulfide bonding and are structurally conserved with Sal1 DBP-II [26].

**Table 1:** Data collection and refinement statistics for DEKnull.

**Data collection**	
Space Group	P2_1_
Cell dimensions	
*a*, *b*, *c* (Å)	55.63, 37.35, 78.20
α, β, γ (◦)	90, 108.77, 90
Resolution (Å)*	20–2.1 (2.2–2.1)
*R*_*sym*_ *	0.116 (0.460)
*I/σI* *	8.51 (2.11)
Completeness (%)*	96.7 (97.5)
Redundancy*	2.9 (2.7)
**Refinement**	
Resolution (Å)	20–2.1
No. reflections	17,535
*R*_*work*_/*R*_*free*_	21.77/25.88
No. atoms^†^	
Protein	2,641
Ligand/ion	0
Water	128
B-factors^†^	
Protein	22.96
Ligand/ion	0
Water	22.59
R.m.s. deviations	
Bond lengths (Å)	0.004
Bond angles (°)	0.749

Data were collected from a single crystal.

*Highest resolution shell is shown in parenthesis

^†^Does not include hydrogens

**Fig 3:**
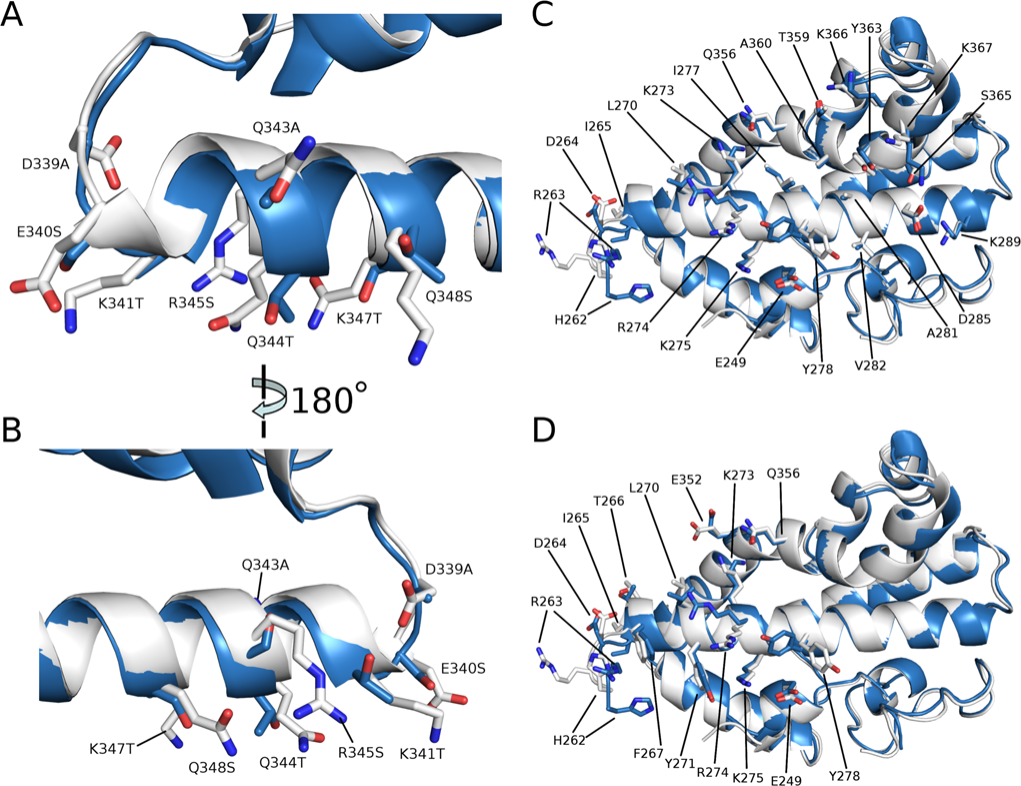
DEKnull mutations do not affect protein secondary structure. (A) and (B) Two views of a structural alignment of Sal1 DBP-II DEKAQQRRKQ polymorphic region (white) and DEKnull ASTAATSRTS mutant region (blue). Mutated residues are labeled and shown as sticks. (C) Structural alignment of DARC binding sites on Sal1 DBP-II (white) and DEKnull (blue). DARC binding residues are labeled and shown as sticks. (D) Structural alignment of DBL dimerization interfaces on Sal1 DBP-II (white) and DEKnull (blue). Dimerization residues are labeled and shown as sticks.

Alignment of DEKnull and Sal1 DBP-II structures shows minimal differences with an overall root-mean-square (r.m.s.) deviation of 0.435 Å (Fig. 2C), indicating there is minimal differences overall between the native and engineered domains. S1 alignment has a r.m.s. deviation of 0.308 Å and is not significantly different (Fig. 2D). S2 alignment has a r.m.s. deviation of 0.288 Å, and the only change is the region comprising K366 to I376, which is now structured in DEKnull as compared to Sal1 DBP-II (Fig. 2D). S3 alignment has a r.m.s. deviation of 0.310 Å and show shifts in loops G417 to D423 and K465 to T473, changes that can be attributed to solvent exposed flexible loops (Fig. 2D). Strikingly, the DEKAQQRRKQ polymorphic stretch within S2 overlaps well between DEKnull and Sal1 DBP-II. Alteration of these amino acids to ASTAATSRTS had no affect on the secondary structure nor do they shift peptide backbone C_α_s (Fig. 3A, 3B).

The dimer interface and DARC binding residues play important roles in host-receptor binding [23,26]. These functional regions are recognized by naturally acquired antibodies that block the DBP-DARC interaction [10,23]. Any DBP-II based synthetic antigen must accurately replicate the three-dimensional conformation of these regions for antibody generation and epitope recognition. We therefore examined if the changes in DEKnull altered these important functional regions. The dimerization and DARC binding surfaces overlap well with the parental Sal1 DBP-II; there is no allosteric change to secondary structure or peptide backbone C_α_s, retaining the conformational shape of protective targets (Fig. 3C, 3D). Furthermore, Define Secondary Structure of Proteins (DSSP) analysis assigns identical secondary structure elements between that of Sal1 DBP-II and DEKnull [59,60]. Together, these structural data demonstrate that the DEKnull conformation is not significantly different from that of the naturally occurring allele, except for the polymorphic DEK epitope, and supports the development of DEKnull as a DBP vaccine.

### Epitope changes in DEKnull

B-cell epitopes fall within two classes: linear and conformational. Linear epitopes are continuous stretches of amino acids in which the primary structure alone is responsible for immunogenicity and antibody recognition. Conformational epitopes can be continuous or discontinuous, but require a fold for immunogenicity and antibody binding. Ablation of the fold through the use of denaturants eliminates antibody recognition of conformational epitopes. While vaccines are able to induce either class, natively folded antigens tend to have a bias towards inducing conformational-dependent antibodies that are protective [61,62]. As a result, it is important to identify and characterize inhibitory and non-inhibitory epitopes on Sal1 DBP-II.

**Fig 4:**
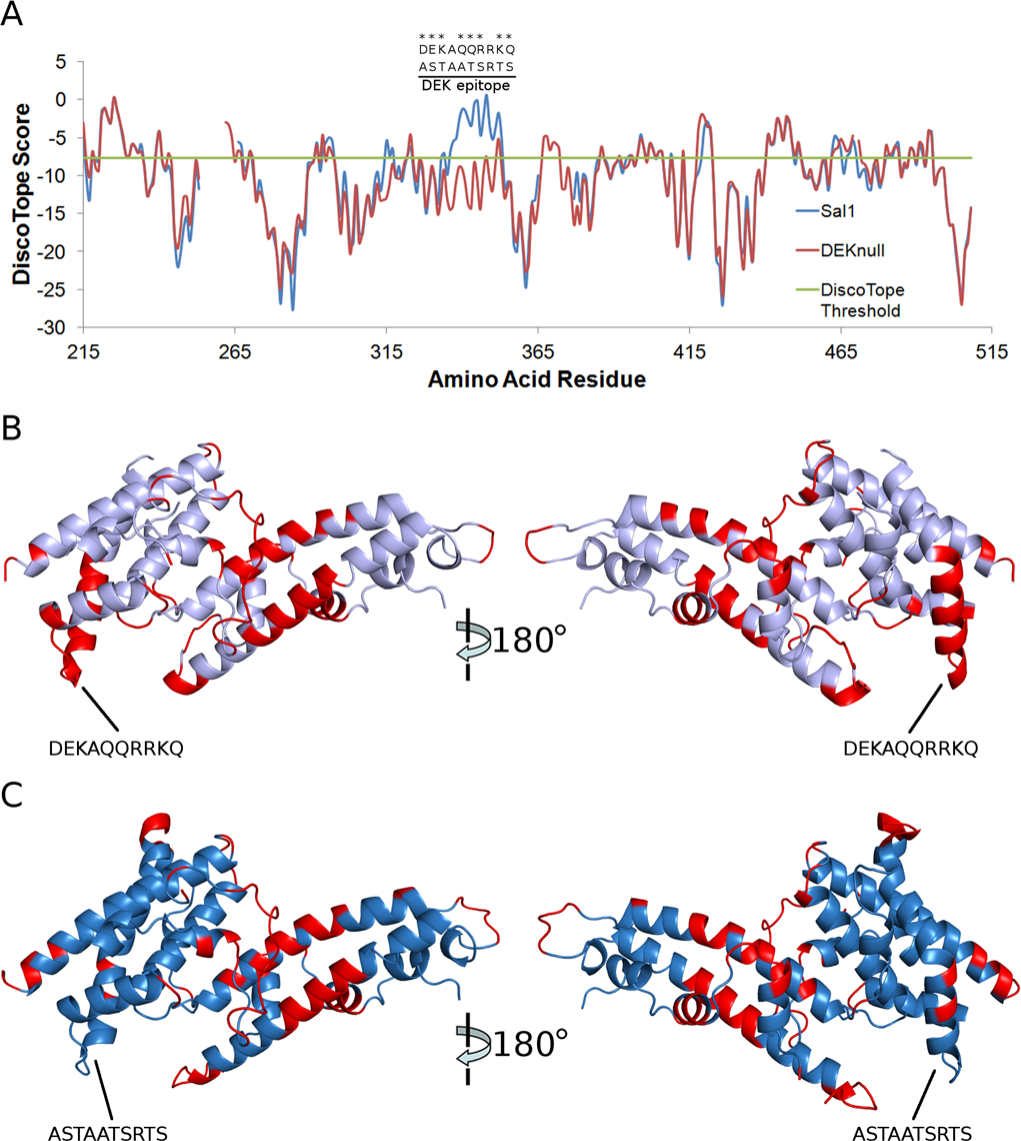
DiscoTope B-cell epitope prediction of Sal1 DBP-II and DEKnull. (A) Graphical representation of DiscoTope B-cell epitope scores for Sal1 DBP-II (blue line) and DEKnull (red line). Prediction threshold is shown in green. DEK residues are located at amino acids 339–348 and shown above the corresponding location in the graph. (B) Two views of Sal1 DBP-II predicted epitopes mapped onto crystal structure. (C) Two views of DEKnull predicted epitopes mapped onto crystal structure.

Bioinformatic B-cell epitope prediction methods for conformational epitopes are a powerful tool that can aid in the rational design and analysis of vaccine targets. DiscoTope is a widely used web-based computational algorithm that focuses on identifying potential discontinuous conformational epitopes based on available crystal structures [63]. DiscoTope analysis of Sal1 DBP-II identifies several distinct epitopes with the strongest signal located at the DEKAQQRRKQ polymorphic patch that is altered within DEKnull (Fig. 4A). The predicted residues are all solvent exposed and are spread across the entire surface of the protein, with no discernible predilection for certain sub-domains (Fig. 4B). DEKnull is predicted to have similar patches of epitopes, but lacks the signal at the DEK location induced by the mutational changes (Fig. 4A, 4C). Comparisons between the Sal1 DBP-II and DEKnull prediction results demonstrate only the DEKAQQRRKQ region is significantly different (Fig. 4A). An important concern of removing decoy-epitopes through mutation is the possibility of introducing novel epitopes caused by the amino acid changes. DiscoTope analysis determines that no new epitopes specific to DEKnull are introduced further demonstrating that DEKnull is a suitable surrogate antigen from native alleles of DBP-II.

### Functional epitopes are conserved in DEKnull

**Fig 5:**
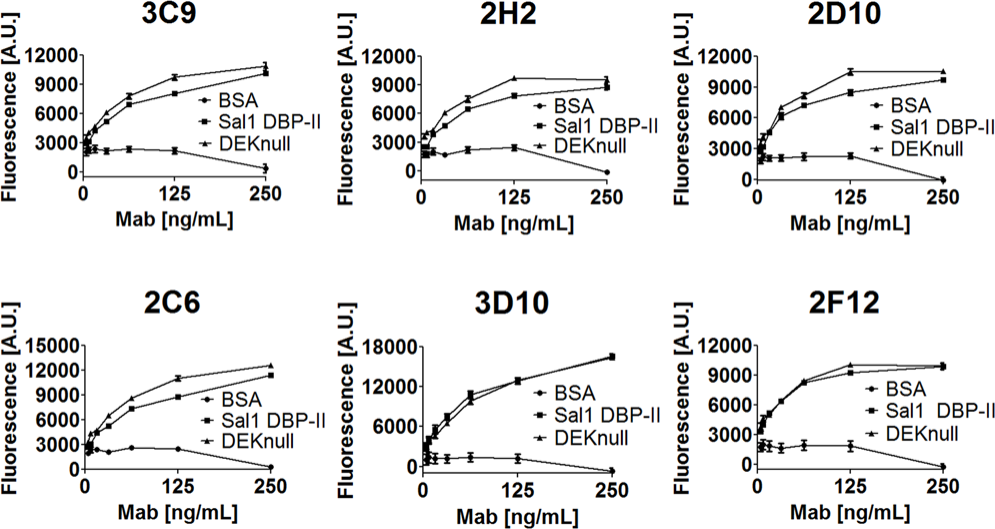
ELISA with anti-DBP conformational specific antibodies. ELISA assays for conformational anti-DBP antibodies with BSA as a negative control, Sal1 DBP-II as a positive control, and DEKnull protein. Four inhibitory (3C9, 2H2, 2C6, 2D10) and two non-inhibitory (3D10, 2F12) antibodies were tested.

The structural and computation approaches indicate that there are no signification changes to epitopes in DEKnull with the exception of the mutated DEKAQQRRKQ epitope. We sought to independently assess the DEKnull antigen retained recognizable epitopes by interrogation with a panel of conformationally dependent anti-Sal1 DBP-II antibodies [47] in ELISA assays. Two non-inhibitory and four inhibitory antibodies were probed; all six antibodies showed no difference in antigen recognition between that of Sal1 DBP-II and DEKnull (Fig. 5). This provides evidence that the DEKnull mutations have minimal effect on the overall structural fold of the protein, and are consistent with the antigenicity results seen in the DiscoTope B-cell epitope prediction (Fig. 4). It is interesting to note that two non-inhibitory antibodies, 3D10 and 2F12, bound to both DBP-II Sal1 and DEKnull equally well (Fig. 5). This suggests that DEKnull still retains at least one other immunogenic region that may continue to function in immune evasion, necessitating further development of DEKnull as a vaccine candidate.

## Discussion

The central role of *P*. *vivax* DBP and the necessity of DARC recognition in parasite invasion during the asexual red blood stage makes it an ideal vaccine target [8]. Anti-DBP antibodies isolated from naturally immune individuals and those generated through immunization are able to prevent DBP-DARC interactions and inhibit parasite growth [6]. However, the inherent polymorphic nature of DBP poses challenges that must be overcome in order to maximize its effectiveness as a vaccine [39,40]. Polymorphic immunodominant epitopes divert the immune system away from weakly immunogenic protective epitopes that are conserved across alleles, resulting in strain-specific responses as opposed to strain-transcending protection [43,45]. This is seen not only with DBP, but is an inherent problem observed with other *Plasmodium* vaccine candidates wherein single allele vaccinations often provide strain-specific inhibition but are yet susceptible to alternate alleles [4,10,64–72].

Currently two parallel strategies exist to enhance DBP as a vaccine candidate and to bypass the issue of polymorphism—a multi-allele vaccine composed of variants found in endemic areas, and a modified vaccine that directs immune responses towards conserved epitopes in order to impart broad protection [46,66,73,74]. The synthetic antigen DEKnull is the brainchild of the latter, an antigen in which a dominant variant B-cell epitope is mutated from the parent Sal1 allele [38]. Vaccination studies with DEKnull demonstrate early proof-of-concept success in manipulating the immune system towards protective responses [5,46]. Further iterations in design are expected to improve immunogenicity, protectivity, and cross-reactivity [5,46].

Here, we present the first structure of DEKnull, a synthetic *Plasmodium* vaccine candidate. These results demonstrate that the DEKnull antigen has insignificant structural changes relative to the parent Sal1 structure [26]. There are virtually no differences in overall DBL fold, orientations of sub-domains 1–3, disulfide bonding, or within the secondary structure and backbone of the mutated region itself (Fig. 2, Fig. 3). The conservation of DBL fold in DEKnull is confirmed with immunological assays examining reactivity against a panel of conformational dependent α-DBP-II(Sal1) antibodies [47]. Of the six antibodies tested, none had significant binding differences between Sal1 and DEKnull (Fig. 5).

The structure of DEKnull additionally allowed us to perform state-of-the-art bioinformatic B-cell epitope analysis through the use of DiscoTope [63]. The prediction results are significant for several reasons. First, the strong signal of the DEK polymorphic patch on the DBP-II Sal1 allele supports that it is strongly immunogenic and can divert immune responses away from conserved protective epitopes. Second, the loss of DEK antigenicity in DEKnull compared to Sal1 further reflects a success in synthetic antigen design in achieving the desired manipulation of epitopes. Third, the DEK mutation did not confound the design of the synthetic antigen by introducing novel epitopes. And finally, the conservation of the remaining epitopes between Sal1 and DEKnull indicates that the mutation does not change the protein’s overall epitope profile suggesting protective epitopes have been retained.

The results first and foremost reflect a success in the strategy of using a modified antigen to bypass DBP allele polymorphisms and poor-protectivity induced by strain-specific epitopes. This study demonstrate that antigen engineering to focus the immune response to conserved functional regions, such as the DARC binding residues and/or DBP dimer interface, is a viable and practical approach. The predicted dominant variant B-cell epitope was eliminated without affecting immunogenicity of the remaining epitopes. Furthermore, the results presented here build upon previous works to establish that protein engineering is a viable approach towards problematic multi-allelic vaccine targets and should guide future vaccine design in other pathogens [5,38].

It has been shown that preliminary immunogenicity studies with DEKnull elicited an immune response comparable to Sal1 DBP-II [5]. A next key step in evaluating DEKnull as a vaccine construct is to demonstrate that DEKnull is able to generate highly potent antibodies that are broadly protective across multiple strains. As a corollary, and one that is predicted *in silico* by DiscoTope results presented here (Fig. 4), DEKnull must also not generate DEKnull-specific antibodies that would be useless against natural alleles.

The ELISA data presented show that DEKnull still possess non-inhibitory epitopes (Mab 3D10 and Mab 2F12, Fig. 5). Characterizing these antibodies will give insight towards the design of future versions of DEKnull. A continual process of eliminating non-protective epitopes from this synthetic antigen will better focus immune responses towards protective targets. Future studies will examine further iterations of DEKnull to improve upon its overall immunogenicity, broad-spectrum inhibitory profile across different *P*. *vivax* DBP alleles, as well as to address the antigenicity of remaining non-protective epitopes.

## Supporting Information

S1 Fig Sequence alignment of Sal1 DBP-II and DEKnull. Sequence alignment of Sal1 DBP-II and DEKnull. The highly polymorphic stretch (DEKAQQRRKQ) is underlined and mutated residues (ASTAATSRTS) in DEKnull are denoted with asterisks (*).
(TIFF)
