# Supplementary figures and images for: Structural Analysis of the Synthetic Duffy Binding Protein (DBP) Antigen DEKnull Relevant for Plasmodium vivax Malaria Vaccine Design
Source: PLoS Negl Trop Dis. 2015 Mar 20;9(3):e0003644. doi: 10.1371/journal.pntd.0003644 (PMC4368114; doi:10.1371/journal.pntd.0003644)

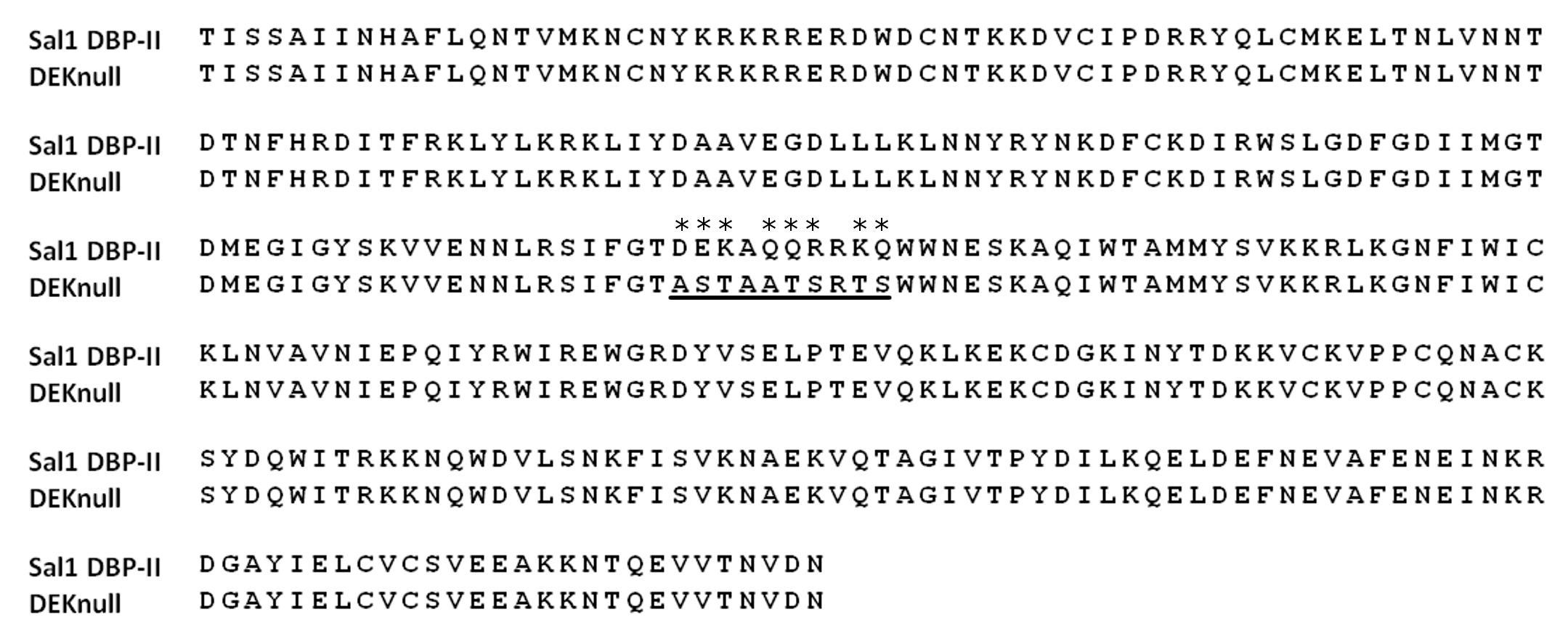

Supplement: S1 Fig — Sequence alignment of Sal1 DBP-II and DEKnull. The highly polymorphic stretch (DEKAQQRRKQ) is underlined and mutated residues (ASTAATSRTS) in DEKnull are denoted with asterisks (*). (TIFF) [file pntd.0003644.s001.tiff]
